# Risk factors for implant failure following revision surgery in breast cancer patients with a previous immediate implant-based breast reconstruction

**DOI:** 10.1007/s10549-020-05911-z

**Published:** 2020-09-12

**Authors:** A. Frisell, J. Lagergren, M. Halle, J. de Boniface

**Affiliations:** 1 Department of Molecular Medicine and Surgery, Karolinska Institutet, 171 77 Solna, Stockholm, Sweden; 2 Department of Surgery, Capio St. Göran’s Hospital, Stockholm, Sweden; 3 Reconstructive Plastic Surgery, Karolinska University Hospital, Stockholm, Sweden

**Keywords:** Implant exchange, Revision surgery, Implant failure, Implant removal

## Abstract

**Purpose:**

The aim of the current study was to evaluate risk factors and timing of revision surgery following immediate implant-based breast reconstruction (IBR).

**Methods:**

This retrospective cohort included women with a previous therapeutic mastectomy and implant-based IBR who had undergone implant revision surgery between 2005 and 2015. Data were collected by medical chart review and registered in the Stockholm Breast Reconstruction Database. The primary endpoint was implant removal due to surgical complications, i.e. implant failure.

**Results:**

The cohort consisted of 475 women with 707 revisions in 542 breasts. Overall, 33 implants were removed due to complications. The implant failure rate (4.7%) was lower without RT (2.4%) compared to RT administered after mastectomy (7.5%) and prior to IBR (6.5%) (*p* = 0.007). While post-mastectomy RT (OR 3.39, 95% CI 1.53–7.53), smoking (OR 3.90, 95% CI 1.76–8.65) and diabetes (OR 5.40, 95% CI 1.05–27.85) were confirmed as risk factors, time from completion of RT (> 9 months, 6–9 months, < 6 months) was not (OR 3.17, 95% CI 0.78–12.80, and OR 0.74, 95% CI 0.20–2.71). Additional risk factors were a previous axillary clearance (OR 4.91, 95% CI 2.09–11.53) and a history of a post-IBR infection (OR 15.52, 95% CI 4.15–58.01, and OR 12.93, 95% CI 3.04–55.12, for oral and intravenous antibiotics, respectively).

**Conclusions:**

Previous axillary clearance and a history of post-IBR infection emerged as novel risk factors for implant failure after revision surgery. While known risk factors were confirmed, time elapsed from RT completion to revision surgery did not influence the outcome in this analysis.

## Introduction

In accordance to national and international guidelines, immediate breast reconstruction (IBR) should be offered [1] when discussing mastectomy with breast cancer patients. The most common surgical method for IBR is implant based, with increasing rates both in Sweden [2] and other countries [3]. One of the most important factors when planning for implant-based IBR is previous or post-mastectomy radiotherapy (PMRT). Radiotherapy (RT) causes chronic inflammatory changes and tissue remodelling [4], and may result in capsular contracture and tissue fibrosis with deteriorated cosmetic outcome, psychological distress and pain as potential complications [5]. As it also negatively affects wound healing and tissue repair [4], it is acknowledged that any further ipsilateral revision surgery entering the implant cavity comes with a higher risk for wound complications and infection. Despite these negative effects, however, it is internationally agreed that PMRT poses no contraindication to IBR in the well-informed patient. In a previous publication from our group, as much as 77.7% of women receiving PMRT would recommend implant-based IBR to other women in their situation, while the corresponding figure was only 68.7% in women with RT prior to IBR [6]. Many women may prefer a potentially temporary implant-based reconstruction, followed by an optional change to an autologous tissue reconstruction, to a simple mastectomy combined with an external prosthesis. Other women may not have sufficient autologous tissue for a delayed reconstruction, and will thus be offered the choice between a skin- or nipple-sparing IBR and a delayed implant-based reconstruction after a simple mastectomy. The latter, however, is fraught with the same negative radiation effects if performed after PMRT.

The timing of implant exchange in the context of PMRT, i.e. replacing an initially placed tissue expander with a permanent fixed-volume implant, has been widely discussed [7, 8]. Furthermore, the time necessary for early irradiation effects to settle before attempting revision surgery, and the association between time from PMRT to revision surgery are as yet debated [9–11]. The aim of this study was to analyse implant failure rates in irradiated versus non-irradiated patients, and identify temporal patterns to define the optimal time frame for scheduling implant revision surgery in the setting of PMRT after implant-based IBR in breast cancer patients.

## Patients and methods

The primary endpoint of this retrospective cohort study was implant removal due to complications after post-IBR revision surgery, i.e. implant failure. To this end, all consecutive patients who had undergone ipsilateral breast implant revision surgery entering the implant cavity, e.g. implant exchange or capsulectomy, at Karolinska University Hospital between 2005 and 2015, and who had previously had a therapeutic mastectomy with implant-based IBR, were identified through their intervention codes. Smaller revision surgeries such as lipofilling, scar correction or nipple reconstruction were disregarded. In a second step, any purely prophylactic mastectomies or incorrectly coded patients were excluded by individual medical chart review. While the use of mesh/acellular dermal matrix (ADM) and prepectoral implant placement have been implemented in Stockholm in later years, there was only prepectoral placement during the studied time period. Only two reconstructions were assisted by ADM, rendering it impossible to draw any conclusions regarding specific associated risks and the impact of RT.

**Fig. 1:**
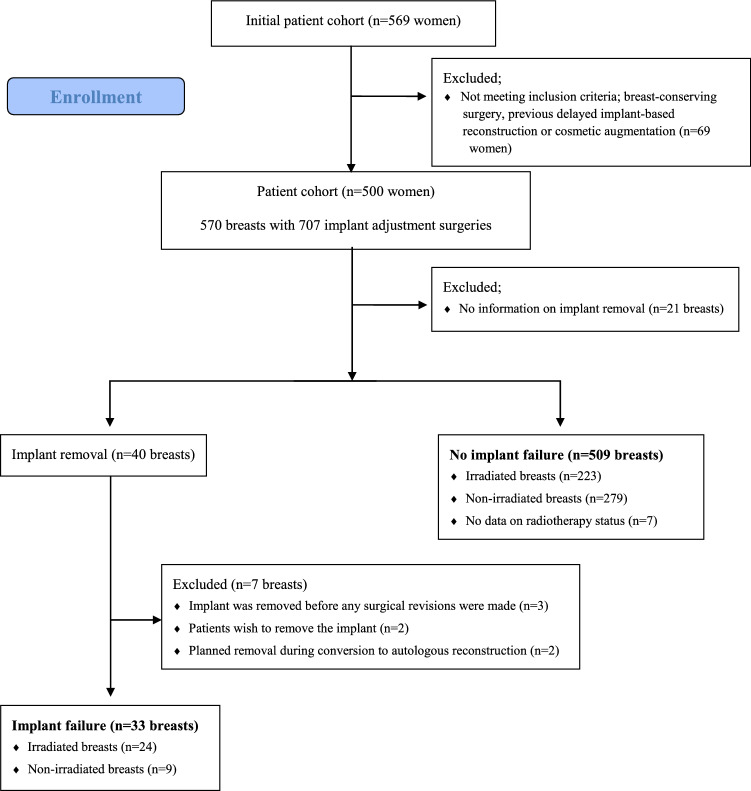
Flow chart for the creation of the final cohort of implant failure (*N* = 33) and no implant failure (*N* = 509) after revision surgery following a previous immediate implant-based breast reconstruction

The aim of this study was to create a decisional aid for patient and surgeon prior to implant revision surgery, focusing on postoperative surgical complications. Patients who had their breast implants removed due to their own wish or during the process of converting an implant-based reconstruction into an autologous reconstruction were removed from the population, in order to avoid any bias regarding each patient´s doctor´s subjective evaluation (see Fig. 1).

Information on tumour characteristics, surgical procedures and oncological treatment with special detail regarding PMRT, complications and lifestyle factors were collected by individual medical chart review for each patient and registered in the retrospective Stockholm Breast Reconstruction Database, which is a pseudonymized digital database constructed in 2017. Cases were censored at the date of implant failure, and the date of the last revision surgery preceding implant failure was used for calculation of time from completion of PMRT to revision surgery as well as time from revision surgery to implant failure.

## Statistical analysis

Categorical data are presented as numbers with their percentages, and continuous variables as median values with their range. For the comparison of categorical variables between the two groups of implant failure and no implant failure, the Chi Square or Fisher’s exact tests, respectively, were used. Comparison of continuous variables in two groups was performed using the Mann–Whitney U test, and in more than two groups using the Kruskal–Wallis test. For tumour and treatment characteristics, data are presented per breast. The association between clinical covariates and the outcome per breast was assessed by univariable binary logistic regression with implant failure as the endpoint, presenting odds ratios and their respective 95% confidence intervals. Multivariable regression analysis was not deemed appropriate due to a low number of observed events. Implant failure rates were calculated per revision surgery in order to mirror the clinical situation of preoperative assessment before the planned surgery.

All data analysis was performed using SPSS® version 24 (IBM, Armonk, New York, USA). Statistical significance was set at the 0.05 level for all analyses.

## Results

After the exclusion of prophylactic and incorrectly registered cases, 475 breast cancer patients (542 breasts) with a previous IBR were identified in whom 707 implant revision surgeries had been performed, see Fig. 1. Median follow-up time, i.e. time from revision surgery to medical chart review, was 95 months (range 2–215). For details on patient and tumour characteristics per breast, see Table 1.

**Table 1:** Tumour and treatment characteristics per operated breast

	Breasts (*n* = 542)
Invasiveness	
In situ only	105 (19.4)
Mixed	277 (51.1)
Invasive only	108 (19.9)
Missing	52 (9.6)
Histopathological tumour size*	
Tis (in situ only)	135 (29.5)
T1 (≤ 20 mm)	55 (12.0)
T2 (21–50 mm)	112 (24.5)
T3/T4 (> 50 mm/locally advanced)	86 (18.8)
Missing	69 (15.2)
Type of invasive cancer	
Ductal	302 (55.7)
Lobular	59 (10.9)
Mixed	7 (1.3)
Other type	4 (0.7)
Missing	170 (31.4)
Histopathological size of largest axillary metastasis*	
None (pN0)	315 (68.9)
ITC (pN0i+)	19 (4.2)
Micrometastasis (pN1mic)	10 (2.2)
Macrometastasis (pN1-3)	103 (22.5)
Missing	10 (2.2)
Chemotherapy**	
Yes	234 (43.2)
No	287 (53.0)
Missing	21 (3.9)
Endocrine therapy**	
Yes	348 (64.2)
No	180 (33.2)
Missing	14 (2.6)
Anti-HER2 therapy**	
Yes	86 (15.9)
No	450 (83.0)
Missing	6 (1.1)

Values in parentheses are percentages unless indicated otherwise

*Patients with neoadjuvant treatment excluded (*n* = 85),

**Received as adjuvant or neoadjuvant treatment

Twenty-four breasts (4.4%) had been irradiated between 1 and 25 years prior to IBR due to a previous breast cancer treated by breast conservation (*N* = 23) or due to lymphoma (*N* = 1). A further 223 breasts (41.1%) had received post-mastectomy RT (PMRT), while 288 breasts (53.1%) had never been irradiated. Information on irradiation was missing for seven breasts. A total of 33 implants (one bilateral case of implant failure) were removed due to surgical complications. Since it is clinically more relevant to know the risk for each surgical intervention, not each breast, the implant failure rate was then calculated per revision surgery and was lowest in non-irradiated breasts (9 out of 375 surgeries, 2.4%) and substantially higher following PMRT (22 out of 293 surgeries, 7.5%) and if RT had been given prior to IBR (2 out of 31 surgeries, 6.5%; overall *p* = 0.007). In most breasts (72.9%), only one revision surgery had been performed, with a median number of revision surgeries entering the implant cavity of one (range 1–5) per breast during follow-up time. The median time from the latest revision to implant failure was 2 months (1–153). Implant failure occurred in 13 cases (8%) if revision surgery had been performed after more than 9 months, in 3 cases (6%) if revision was undertaken after 6–9 months, and in 3 (21.4%) of women where it was done after less than 6 months from completion of RT. Probably due to the low event rate and few cases with short time between RT completion and first revision surgery, this difference was not statistically significant (*p* = 0.171; see Table 2). Unadjusted risk factors for implant failure, calculated per breast, are presented in Table 3. Known risk factors such as current smoking and diabetes were confirmed in the present cohort. Interestingly, postoperative infection reported after the initial IBR increased the risk of implant failure after revision surgery significantly, whether the previous infection was diagnosed based on suspicious clinical signs only (*p* = 0.005) or confirmed by bacterial cultures and/or elevated inflammatory markers such as the C-reactive protein (CRP) (*p* < 0.001). This is noteworthy since these infections had obviously not resulted in an implant failure during the postoperative period following IBR. As expected, PMRT was a significant risk factor for implant failure. Looking at the RT target, however, risk for implant removal was only increased if locoregional RT was given, not if it only targeted the chest wall. Likewise, the performance of an axillary clearance, intimately associated with node positivity, increased the risk fivefold. In order to differentiate between the effect of locoregional RT and that of axillary clearance, both covariates were entered into the same regression model: Here, locoregional RT lost its significance (OR 2.11, 95% CI 0.85–5.24) when compared with no RT, while axillary clearance retained its significant negative effect on the risk of implant failure (OR 2.99, 95% CI 1.08–8.27).

**Table 2:** Radiotherapy details per operated breast (*N* = 542)

	Implant failure (*n* = 33)	No implant failure (*n* = 509)	*p*
Radiotherapy			0.007
Yes, prior to IBR	2 (8.3)	22 (91.7)	
Yes, after IBR	22 (9.9)	201 (90.1)	
None	9 (3.1)	279 (96.9)	
Missing	0	7	
Type of adjuvant radiotherapy*			0.018
Local	3 (3.7)	79 (96.3)	
Locoregional	19 (13.5)	122 (86.5)	
Missing	0	0	
Radiotherapy dose/fractions*			0.667
46 Gy/23 fractions	2 (11.1)	16 (88.9)	
50 Gy/25 fractions	20 (10.1)	178 (89.9)	
Other	0	7	
Missing	0	0	
Months from end of radiotherapy to first surgical revision			
> 9 months	13 (8.0)	150 (92.0)	0.171
6–9 months	3 (6.0)	47 (94.0)	
< 6 months	3 (21.4)	11 (78.6)	
Missing	5	15	

*IBR* immediate implant-based breast reconstruction

*Patients who had received other than adjuvant RT after IBR were excluded

**Table 3:** Univariable logistic regression analysis with implant failure after revision surgery as the endpoint

	Univariable			
	All cases	Implant failure	Odds ratio	*p*
Total number of breasts	542	33		
Age (years) at IBR				
< 50	319	21	1.00 (reference)	
50–60	163	10	0.93 (0.43–2.02)	0.850
> 60	60	2	0.49 (0.11–2.14)	0.343
Missing	0	0		
Histopathological tumour stage				
Tis (in situ only)	135	11	1.00 (reference)	
T1 (≤ 20 mm)	55	1	0.21 (0.03–1.66)	0.138
T2 (21–50 mm)	112	4	0.42 (0.13–1.35)	0.144
T3/T4 (> 50 mm)	86	5	0.70 (0.23–2.08	0.516
Missing	154	12		
Histopathological nodal stage				
Node negative	407	17	1.00 (reference)	
Node positive	111	14	3.31 (1.58–6.95)	0.002
Missing	24	2		
Axillary clearance				
No	290	7	1.00 (reference)	
Yes	240	26	4.91 (2.09–11.53)	< 0.001
Missing	12	0		
Type of implant				
Temporary expander	33	1	1.00 (reference)	
Permanent expander	434	29	2.29 (0.30–17.37)	0.422
Fixed-volume implant	68	2	0.97 (0.09–11.10)	0.980
Missing	7	1		
Final implant volume*				
< 300 cc	151	8	1.00 (reference)	
300–400 cc	265	12	0.85 (0.34–2.12)	0.724
> 400 cc	118	10	1.66 (0.63–4.33)	0.305
Missing	8	3		
Radiotherapy				
None	288	9	1.00 (reference)	
Yes, prior to IBR	24	2	2.82 (0.57–13.85)	0.202
Yes, after IBR	223	22	3.39 (1.53–7.53)	0.003
Missing	7	0		
Type of adjuvant radiotherapy**				
Local	82	3	1.00 (reference)	
Locoregional	141	19	4.10 (1.18–14.32)	0.027
Missing	0	0		
Radiotherapy dose/fractions**				
46 Gy/23 fractions	18	2	1.00 (reference)	
50 Gy/25 fractions	198	20	0.90 (0.19–4.20)	0.892
Missing/other	7	0		
Months from end of radiotherapy to first surgical revision				
> 9 months	163	13	1.00 (reference)	
6–9 months	50	3	3.17 (0.78–12.80)	0.106
< 6 months	14	3	0.74 (0.20–2.71)	0.651
Missing	20	5		
Postoperative infection within 30 days after IBR				
No infection	398	10	1.00 (reference)	
Clinical signs of infection, oral antibiotic treatment	72	7	4.18 (1.54–11.37)	0.005
Confirmed infection, oral antibiotic treatment^a^	14	4	15.52 (4.15–58.01)	< 0.001
Confirmed infection, intravenous antibiotic treatment^a^	12	3	12.93 (3.04–55.12)	0.001
Missing	46	9		
Postoperative complication with return to theatre (within 30 days) after IBR				
No	489	24	1.00 (reference)	
Yes	11	1	1.94 (0.24–15.76)	0.536
Missing	42	8		
Postoperative complication without return to theatre after IBR				
None	464	23	1.00 (reference)	
Seroma	44	4	1.92 (0.63–5.82)	0.250
Infection	5	2	12.78 (2.04–80.30	0.007
Bleeding	8	1	2.74 (0.32–23.21)	0.355
Skin necrosis	7	1	3.20 (0.37–27.66)	0.291
≥ 2 complications	14	2	3.20 (0.68–15.13)	0.143
Missing	0	0		
Previous revision surgery performed				
No	395	22	1.00 (reference)	
Yes	147	11	1.37 (0.65–2.90)	0.409
Missing	0	0		
BMI^c^				
Normal (18.5–30)	438	18	1.00 (reference)	
Underweight (< 18.5)	8	1	3.33 (0.39–28.55)	0.272
Overweight (> 30)	23	3	3.50 (0.95–12–87)	0.059
Missing	73	11		
Smoking^c^				
Never smoked	383	19	1.00 (reference)	
Currently smoking	65	11	3.90 (1.76–8.65)	0.001
Former smoker	73	2	0.54 (0.12–2.37)	0.414
Missing	21	1		
Immunosuppressive treatment^c^				
No	498	31	1.00 (reference)	
Yes	9	2	3.57 (0.74–17.24)	0.113
Missing	35	0		
Antihypertensive medication^c^				
No	454	26	1.00 (reference)	
Yes	42	5	2.08 (0.76–5.70)	0.155
Missing	46	2		
Diabetes^c^				
No	502	31	1.00 (reference)	
Yes, with medication^b^	6	2	5.40 (1.05–27.85)	0.044
Missing	34	0		

Each case represents one operated breast

*IBR* immediate implant-based breast reconstruction

*Final expander volume or fixed-volume implant size

**Reporting only patients who had received RT after IBR

^a^Confirmed by positive bacterial cultures and/or elevated C-reactive protein

^b^Including oral medication and/or insulin

^c^Registered at the time of IBR, not at implant revision surgery

## Discussion

The optimal timing of revision surgery after IBR, especially in irradiated patients, remains a question of clinical assessment for most surgeons. While irradiation posed a significant risk for implant failure following revision surgery, we could not find support for a clinical role of timing of the revision surgery in relation to completed RT, probably because of few events and a generally long interval between RT and first revision surgery. Additional factors like the irradiated target volume, previous axillary clearance and a history of infection after IBR appear important to take into consideration.

In Sweden, revision surgery entering the implant cavity after a previous IBR is only undertaken once RT has been completed, and a minimum time of 6 months between RT completion and revision surgery has long been recommended. Santosa et al. showed that there was no difference in complication rates among women who received PMRT onto an expander or after the exchange to a permanent implant [12]. For women without indications for RT, revision surgery (most commonly an exchange of the implant) can be performed as early as 1 month after full expansion of the expander device [11, 13]. The current recommendations of exchange to a permanent implant in the setting of PMRT range from 3 to 6 months after RT [8, 14]. However, Peled et al. showed that the implant failure rate significantly decreased from 22.4 to 7.7% (*p* = 0.036) if more than 6 months elapsed between the completion of PMRT and the exchange procedure [8]. Noteworthy, the current study is one of few studies conducted in a non-two-stage breast reconstruction setting with the aim to evaluate temporal aspects of revision following RT. More time has therefore passed between completion of RT and revision surgery and comparison is thus difficult. Especially Peled et al. compared very early implant exchange (3 months) with 6 months (at a median of 14 weeks and 37 weeks, respectively) after completion of RT in a planned two-stage breast reconstruction setting. In the present cohort, however, a majority of the patients received a permanent expander without any timeline for a planned implant exchange, where the influence of time seems to be less important, unlike many of the centres that have studied a planned revision in a two-stage setting.

Axillary treatment, in Sweden including both axillary clearance and locoregional radiotherapy in patients with any axillary macrometastases outside of clinical trials, significantly increased the risk of implant failure. Interestingly, it was the axillary surgery that carried the significant increase in risk when adjusted for locoregional RT. In agreement with this, long-term negative consequences such as arm lymphoedema were significantly stronger associated with axillary surgery than with locoregional RT [15]. It is likely that scarring and fibrotic changes in the axilla decrease lymphatic drainage from the remaining chest wall, leading to delayed wound healing and an enhanced susceptibility to infection [16].

Our results show that even a transient clinically diagnosed infection after the initial IBR was a strong predictor of implant failure after the revision procedure. This is probably due to residual subclinical infection despite clinically successful antibiotic treatment, inducing capsular contraction [17]; remaining subclinical bacterial presence in capsular tissue or the implant coating itself may be re-activated during revision surgery, possibly explaining the increased risk for implant loss such a long time after IBR itself. While the cumulative infection rate was high, it needs to be taken into account that even the slightest suspicion of infection, without any supporting proofs, was considered. In such cases, antibiotics may well only be administered for sheer safety since an unacknowledged infection would have devastating consequences on the implant reconstruction. Unfortunately, there was no confirmative information regarding new clinical signs of infection after revision surgery, which limits the strength of this hypothesis. It would be interesting to test the hypothesis whether prolonged prophylactic antibiotic usage in the setting of high-risk revision surgery could decrease implant failure rates.

Limitations of the study need to be acknowledged. First, in retrospective studies, there is a limitation in the extent of information registered in medical charts. Although our patient cohort is relatively large, the number of events, i.e. implant failures, was surprisingly low, which made advanced statistical adjustments unfeasible. On the other hand, this same fact should underline that revision surgery is a safe procedure even in the face of a number of risk factors identified. Second, since we only selected implant removal due to surgical complications but not due to discomfort, inferior cosmetic results or patient wish (often resulting in the conversion of an implant-based reconstruction to an autologous method), the rate of implant removal may have been underestimated. While the present analysis focussed on surgical complications as the reason for implant failure, we will in an upcoming analysis report on rates of conversion to autologous breast reconstruction, which can well be counted towards implant failure, too. Third, clinical factors such as postoperative complications, BMI and smoking were registered for IBR, not for the subsequent implant revision surgery. Likewise, all included patients had at least one revision surgery, and thus, women who opted for an autologous conversion without any previous attempt at revision were not registered. Since these alternative outcomes also carry significant clinical importance, the overall implant removal should be further explored in future analyses.

## Data Availability

The datasets generated and/or analysed during the current study are not publicly available due to confidentiality reasons regulated by Swedish law. Data can be requested from the register holder.
